# Enhanced Synaptic Properties in Biocompatible Casein Electrolyte via Microwave-Assisted Efficient Solution Synthesis

**DOI:** 10.3390/polym15020293

**Published:** 2023-01-06

**Authors:** Hwi-Su Kim, Hamin Park, Won-Ju Cho

**Affiliations:** 1 Department of Electronic Materials Engineering, Kwangwoon University, Gwangun-ro 20, Nowon-gu, Seoul 01897, Republic of Korea; 2 Department of Electronic Engineering, Kwangwoon University, Gwangun-ro 20, Nowon-gu, Seoul 01897, Republic of Korea

**Keywords:** microwave, biocompatible polymers, casein, electric double layer, synaptic transistors, artificial synapses, neuromorphic computing

## Abstract

In this study, we fabricated an electric double-layer transistor (EDLT), a synaptic device, by preparing a casein biopolymer electrolyte solution using an efficient microwave-assisted synthesis to replace the conventional heating (heat stirrer) synthesis. Microwave irradiation (MWI) is more efficient in transferring energy to materials than heat stirrer, which significantly reduces the preparation time for casein electrolytes. The capacitance–frequency characteristics of metal–insulator–metal configurations applying the casein electrolyte prepared through MWI or a heat stirrer were measured. The capacitance of the MWI synthetic casein was 3.58 μF/cm^2^ at 1 Hz, which was higher than that of the heat stirrer (1.78 μF/cm^2^), confirming a stronger EDL gating effect. Electrolyte-gated EDLTs using two different casein electrolytes as gate-insulating films were fabricated. The MWI synthetic casein exhibited superior EDLT electrical characteristics compared to the heat stirrer. Meanwhile, essential synaptic functions, including excitatory post-synaptic current, paired-pulse facilitation, signal filtering, and potentiation/depression, were successfully demonstrated in both EDLTs. However, MWI synthetic casein electrolyte-gated EDLT showed higher synaptic facilitation than the heat stirrer. Furthermore, we performed an MNIST handwritten-digit-recognition task using a multilayer artificial neural network and MWI synthetic casein EDLT achieved a higher recognition rate of 91.24%. The results suggest that microwave-assisted casein solution synthesis is an effective method for realizing biocompatible neuromorphic systems.

## 1. Introduction

The human brain has the ability to simultaneously calculate and memorize complex information with an ultra-low energy consumption of ~20 W [1]. This high-efficiency biological computing is accomplished through massive parallel processing, fault tolerance, and self-learning through a nervous system with ~10^11^ neurons and ~10^15^ synapses [2,3]. Classical computing systems based on the von Neumann architecture, unlike the human brain, faced increasingly higher power consumption and low computing speed limitations in terms of information processing due to bottlenecks between physically separated processors and memory units [4,5,6]. To overcome these limitations, neuromorphic systems, an innovative computing architecture inspired by the exceptional performance of the human brain in hardware, have received significant attention [7,8]. For implementing an efficient neuromorphic computing system, analog electronics that mimic synaptic functions, including two- and three-terminal devices, are key. This is because analog synaptic devices can analogically modulate the conductance of devices such as biological synaptic plasticity [9,10].

Two-terminal synaptic devices, such as memristor [11,12,13], spintronics device [14], and phase-change device [15,16,17], have been proposed through neural operation and geometrical advantages [8,18]. However, unlike the human-brain system, the two-terminal devices have limitations in that learning and signaling are performed separately. This is because the terminal receives feedback from post-neurons in the learning process and outputs signals from pre-neurons for signal transmission [19]. This limitation represents an incomplete implementation of biological synapses in two-terminal devices. In contrast, a three-terminal synaptic device can simultaneously learn and transmit signals through different parts, the gate terminal and the channel, respectively [20]. As a result, synaptic behavior can be fully emulated by a three-terminal device. In recent years, three-terminal synaptic transistors using ion-conducting electrolytes as gate insulators have been studied. Ion-conducting electrolytes contribute to electric double-layer (EDL) formation, allowing the synaptic function of electric double-layer transistors (EDLTs) to be achieved [21,22]. EDLs, which act as nanogap capacitors, have large capacitances (>1 μF/cm^2^), resulting in very strong gate coupling between interfacial electrons/ions in EDLTs [22,23,24]. Thus, EDLTs can control synaptic plasticity with low power through channel conductance modulated by ion migration inside the electrolyte in response to gate bias [25,26]. In addition, as interest in eco-friendly and biocompatible electronic devices increases, biomaterials based on polymers, such as chitosan [27,28], starch [29,30], gelatin [31,32], and pectin [33], have been applied to EDLTs. Recently, it has been reported that casein, which is a biopolymer accounting for 80% of milk protein, has been utilized for EDLTs mimicking synaptic behavior based on its rich internal protons [34]. Moreover, these electrolytes are abundant in nature and inexpensive, especially in solution-based processes [28].

Meanwhile, chemical synthesis through microwave irradiation (MWI) was first reported in 1986 [35]. The polar molecules in the reaction mixture react quickly to the rapidly changing electric field of the MWI. As a result, rotation and friction of the polar molecules are induced, enabling direct and homogeneous internal heating in the reaction mixture [36,37]. Therefore, until now, MWI has been spotlighted and applied to various organic synthesis and material preparation due to its feature of being an environmentally friendly high-efficiency heating method [38,39]. Furthermore, MWI has several advantages over conventional heating methods, such as faster heating speed, faster processing time, volumetric heating, controllable heating, higher heat transfer efficiency, low energy consumption, and cost-effective processing [40,41,42].

In this study, we prepared a casein biopolymer electrolyte solution using efficient microwave heating by replacing the conventional heat stirrer and implemented improved synaptic properties through casein electrolyte-based EDLTs prepared by microwave-assisted solution synthesis. Prior to the fabrication of the MWI synthetic casein electrolyte-gated EDLTs, the frequency-dependent capacitance of metal–insulator–metal (MIM) capacitors with indium tin oxide (ITO) electrodes and casein electrolyte (synthesized through MWI) was evaluated to verify the effect of the EDL gating effect in the MWI casein electrolyte. By constructing our proposed EDLTs, essential functions related to synaptic plasticity, including excitatory post-synaptic current (EPSC), paired-pulse facilitation (PPF), signal filtering, and potentiation/depression, were evaluated. In addition, we performed recognition simulations using the Modified National Institute of Standards and Technology (MNIST) dataset via a multilayer artificial neural network (ANN) to demonstrate the application of the proposed EDLTs in neuromorphic systems. The outcomes were compared with those of EDLTs gated by the heat stirrer synthetic casein electrolyte. The notable findings of this study are, firstly, a significant reduction in casein electrolyte synthesis time through MWI heating; secondly, enhanced synaptic properties based on higher facilitation; and, thirdly, the improved recognition rate of MWI casein EDLTs.

## 2. Materials and Methods

### 2.1. Fabrication of Devices

We fabricated casein electrolyte-gated EDLTs on glass substrates (Corning Inc., New York, NY, USA) using either MWI synthesis or heat-stirrer synthesis approaches. To form the bottom-gate electrode, a 300 nm-thick ITO film (In_2_O_3_:SnO_2_ = 9:1 mol%, THIFINE Co., Ltd., Incheon, Republic of Korea) was deposited on the glass substrate via radio frequency (RF) magnetron sputtering. The RF power, chamber pressure, and Ar flow of ITO sputtering were 100 W, 3 mTorr, and 20 sccm, respectively. To prepare two types of casein solutions, 3 wt.% of casein powder (technical grade, Sigma-Aldrich, St. Louis, MO, USA) and 3 wt.% of acetic acid (purity > 99%, Sigma-Aldrich) were dissolved in 94 wt.% of deionized water using the MWI or conventional heat-stirrer synthesis method. For the MWI synthesis process, a microwave of frequency 2.45 GHz and power 250 W was irradiated for 5 min. Meanwhile, a magnetic heat stirring of 800 rpm was performed at 130 °C for 6 h for the heat-stirrer synthesis process. These synthesized precursor solutions were filtered through a 5 μm-pore-size polytetrafluoroethylene syringe filter to remove contaminants. Then, each casein solution was spin-coated on an ITO/glass substrate at 3000 rpm for 30 s and dried in air for 24 h. An indium gallium zinc oxide (IGZO) film (In_2_O_3_:Ga_2_O_3_:ZnO = 4:2:4.1 mol%, THIFINE Co., Ltd.) with a thickness of 50 nm was deposited through a shadow mask using RF magnetron sputtering to form a transistor channel layer with dimensions (width × length) 1000 μm × 80 μm. Finally, 150 nm-thick ITO source/drain electrodes with dimensions 1000 μm × 200 μm were deposited through a shadow mask using RF magnetron sputtering.

### 2.2. Characterizations of the Devices

The fabricated casein electrolyte-gated EDLTs were placed in a dark box to block external light and electrical noise. The capacitance versus frequency (C–*f*) characteristic curves of ITO/casein electrolyte/ITO capacitors were analyzed using the Agilent 4284A precision LCR meter (Hewlett-Packard Corp., Palo Alto, CA, USA). The electrical properties and synaptic functions of the casein electrolyte-gated EDLTs were characterized using an Agilent 4156B precision semiconductor parameter analyzer (Hewlett-Packard Corp., USA). In addition, the Agilent 8110A pulse generator (Hewlett-Packard Corp., USA) was used to apply electrical pre-synaptic stimuli to validate the synaptic behavior of the fabricated EDLTs.

## 3. Results

### 3.1. Preparation of a Casein Electrolyte Solution

**Figure 1:**
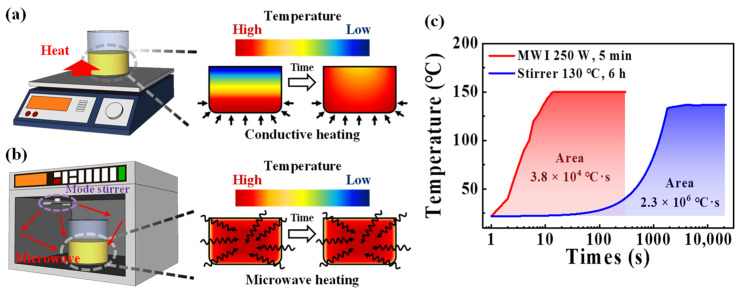
Schematic illustration of solution synthesis equipment and internal temperature profile of casein electrolyte solution for (**a**) heat-stirrer and (**b**) microwave irradiation (MWI) synthetic processes. (**c**) Temperature profiles and thermal budgets of conventional heat stirrer (130 °C, 6 h) and MWI (250 W, 5 min) processes for preparing casein electrolyte solution.

Figure 1a,b show diagrams of the synthesis equipment and internal temperature profiles of casein electrolytes for heat-stirrer and MWI processes, respectively. In the conventional heat-stirrer synthesis, the solution temperature has a non-uniform temperature distribution where the bottom of the vessel is the highest while the top surface is the lowest. Therefore, a considerable period of time is required until the temperature difference decreases due to conduction and convection phenomena. In contrast, since MWI transfers electromagnetic energy directly to the solution, the uniform temperature distribution is achieved by volumetric heating that occurs simultaneously with the initiation of the synthesis process. The temperature profiles of the casein precursor solution during the heat-stirrer and MWI synthesis processes are shown in Figure 1c. The temperatures of the solution measured using an infrared (IR) thermometer were 135 °C for the heat stirrer and 150 °C for the MWI. The thermal budgets for both processes were obtained by integrating the corresponding temperature profiles with respect to the processing times. The conventional heat-stirrer synthesis process was performed at 130 °C for 6 h and had a thermal budget of 2.3 × 10^6^ °C·s. The MWI synthesis process was performed at 250 W for 5 min and had a thermal budget of 3.8 × 10^4^ °C·s. Consequently, the MWI process is more efficient in casein electrolyte synthesis because it takes less time and has 82-times lower thermal budget than a heat-stirrer process.

### 3.2. EDL Operation of MWI Synthetic Casein Electrolytes

**Figure 2:**
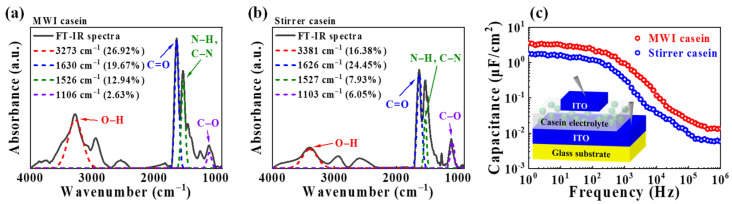
FT-IR spectra of casein electrolyte films produced through (**a**) MWI (250 W, 5 min) and (**b**) heat-stirrer (130 °C, 6 h) processes. (**c**) Frequency-dependent specific capacitance of capacitors using casein electrolyte films synthesized by MWI or heat-stirrer processes. Inset: schematic illustration of the measured ITO/casein electrolyte/ITO vertical sandwich structure.

To identify the difference in the chemical properties of the spin-coated casein electrolyte film prepared through MWI or heat-stirrer synthesis, Fourier-transform infrared spectroscopy (FT-IR) analysis was executed in a range of 900 to 4000 cm^−1^, as shown in Figure 2a,b. Broad peaks at approximately 3273 and 3381 cm^−1^ in MWI and heat-stirrer synthetic casein electrolytes, respectively, are attributed to O–H stretching [43]. In particular, casein, which is abundant in mobile protons, is a protein substance that accounts for about 80% of the total protein in milk, and the main component of the protein is an amide group [44]. Therefore, peaks related to the amide group were observed from 1700 to 1500 cm^−1^ in both casein electrolyte films, in which the peaks from 1700 to 1600 cm^−1^ and 1600 to 1500 cm^−1^ corresponded to the amide I and amide II groups, respectively. The peaks at 1630 and 1626 cm^−1^ in MWI and heat-stirrer synthetic casein electrolytes, respectively, are caused by C=O stretching. The peaks at 1526 and 1527 cm^−1^ in the MWI and heat-stirrer synthetic casein electrolytes, respectively, are due to N–H bending and C–N stretching. In addition, peaks near 1106 and 1103 cm^−1^ in MWI and heat-stirrer synthetic casein electrolytes, respectively, are associated with C–O stretching [45,46]. For quantitative analysis of the peaks, peak deconvolution was performed and the peak area was divided by the total spectra area to obtain a percentage of the peak area [47,48]. The area percentages of the O–H, amide I, amide II, and C–O related peaks were 26.92, 19.67, 12.94, and 2.63%, respectively, for the MWI synthetic casein electrolyte, while they were 16.38, 24.45, 7.93, and 6.05% in the heat-stirrer synthetic casein electrolyte, respectively. The presence of O–H groups could help with proton conduction, which facilitates the formation of EDL by promoting proton migration within the casein electrolyte [29]. Therefore, the MWI synthetic casein electrolyte has more O–H groups than heat-stirrer synthetic casein electrolyte, leading to higher ionic conduction. MWI is a way to facilitate chemical reactions [49], and microwaves loosen the structure of protein molecules, exposing more active groups to the synthesis process [50]. Therefore, more chemical reactions occur for the MWI synthesis process of casein solution, resulting in a higher O–H group ratio in the MWI casein electrolyte. Subsequently, MIM capacitors with a vertical sandwich structure were fabricated using casein electrolytes as an insulating layer and ITO as a metal layer to verify the EDL gating effect in synthetic casein electrolytes. Figure 2c shows the frequency-dependent capacitance characteristics over a wide frequency range from 1 Hz to 1 MHz. The maximum capacitance appeared at 1 Hz and the capacitance decreased with increasing frequency. This change is due to the difference in the response time of the mobile protons in the casein electrolyte according to the frequency. At low frequencies, protons accumulate at the two interfaces (electrolyte/channel and electrolyte/gate) to form an EDL because protons have a sufficient response time to reach the interface. The result is a huge capacitance due to the formed EDL, which is a nanometer-thick parallel capacitor [51]. It is noteworthy that the maximum capacitance of the MWI synthetic casein electrolyte was 3.58 μF/cm^2^, which was higher than that of the heat-stirrer synthetic casein electrolyte (1.78 μF/cm^2^). The large capacitance of the EDL has a strong gating effect between the channel and the gate, allowing for tuning of the conductivity, even at low voltages [22]. Therefore, more energy-efficient artificial synapses can be implemented using the MWI synthetic casein electrolyte-gated EDLT.

### 3.3. MWI Synthetic Casein Electrolyte-Gated EDLT

**Figure 3:**
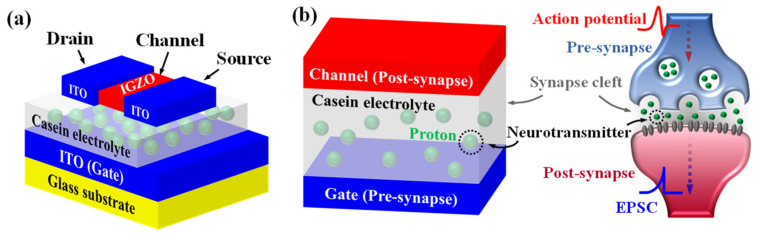
(**a**) Schematic of the proposed microwave-assisted synthetic casein electrolyte-gated electric double-layer transistor (EDLT). (**b**) Comparison between the functional mechanism of the proposed synaptic transistor and biological synapses.

Figure 3a shows the structure of the proposed microwave-assisted synthetic casein electrolyte-gated EDLT. A structural comparison between the functional mechanisms of the proposed EDLT and biological synapses is shown in Figure 3b. The gate electrode and channel layer of the EDLT are considered pre- and post-synapse, respectively. The casein electrolyte between the gate and the channel is considered a synaptic cleft. In biological synapses, changes in synaptic weight are caused by the transmission of neurotransmitters, including K^+^ and Na^+^ ions, through the synaptic cleft, which is analogous to the channel conductance changes in the EDLT induced by proton transport of casein electrolytes. Therefore, the drain current associated with the channel conductance is indicative of EPSC and its change is indicative of synaptic plasticity.

### 3.4. Electrical Properties of Devices

**Figure 4:**
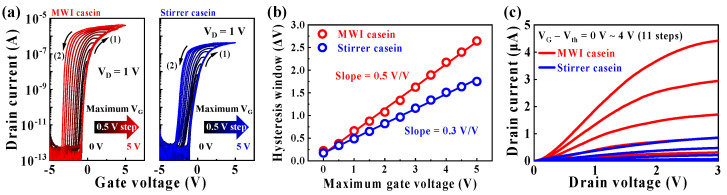
(**a**) Double-sweep transfer characteristic curves of MWI and heat-stirrer synthetic casein electrolyte-gate EDLTs as a function of gate voltage sweep range (maximum V_G_ from 0 V to 5 V in 0.5 V increments) at a V_D_ of 1 V. (**b**) Extracted hysteresis window as a function of maximum V_G_. (**c**) Output characteristic curves of MWI and heat-stirrer synthetic casein electrolyte-gated EDLTs versus gate voltage—threshold voltage (V_G_ − V_th_ = 0–4 V in 0.4 V increments).

Figure 4a shows the transfer characteristic curves (I_D_–V_G_) of MWI and heat-stirrer synthetic casein electrolyte-gate EDLTs measured in double-sweep mode of gate voltage (V_G_). The V_G_ double-sweep as the backward after the forward sweeps was performed at a drain voltage (V_D_) of 1 V, increasing the maximum V_G_ from 0 to 5 V in 0.5 V increments. In the double-sweep transfer curve, the direction of the hysteresis window exhibits a counterclockwise shape and the hysteresis window increases with increasing maximum V_G_ sweep range. This hysteresis characteristic is due to the slow polarization of mobile protons in the casein electrolyte. In the V_G_ forward sweep, the larger the maximum V_G_, the more protons are induced at the interface of the casein electrolyte/IGZO channel. Then, in the V_G_ backward sweep, the protons progressively diffuse back in the opposite direction, increasing the counterclockwise hysteresis window. Figure 4b shows the hysteresis window (ΔV) according to the maximum V_G_ obtained from the double-sweep transfer characteristic curves. The ΔV expanded from 0.22 V to 2.64 V with a 0.5 V/V slope in the MWI synthetic casein EDLT, indicating a greater increase in the ΔV than that of the heat stirrer (from 0.17 V to 1.75 V with a 0.3 V/V slope). Figure 4c shows the output characteristic curve (I_D_–V_D_), where the drain current (I_D_) increases linearly as V_D_ increases, followed by pinch-off and saturation characteristics. Since the MWI synthetic casein electrolyte has a stronger EDL gate effect, the MWI synthetic casein EDLT exhibits larger drain current and ΔV slope than that of the heat stirrer.

Table 1 summarizes the electrical parameters of MWI and heat-stirrer synthetic casein EDLTs extracted from transfer characteristic curves at a maximum V_G_ of 5 V. The MWI synthetic casein EDLTs exhibited improved transfer properties compared to the heat-stirrer synthetic casein electrolyte-gated EDLTs, with higher on/off current ratio (I_on_/I_off_), field-effect mobility (μ_FE_), and hysteresis window (ΔV), but lower threshold voltage (V_th_), subthreshold swing (SS), and interface trap density (D_it_). In particular, the higher μ_FE_ and lower SS are attributed to the better interfacial state, lower root-mean-square surface roughness, and D_it_ values of MWI synthetic casein. The thickness and surface roughness of the coated films are shown in Figure S1 (Supplementary Materials).

### 3.5. Synaptic Properties of Devices

In the human brain, biological signals are transmitted by diffusing the neurotransmitters between pre- and post-synapses, and in this process, EPSCs, which are transient current, are generated. The EPSC represents the synaptic weight regulated by the ion flux [28,52], which increases as the duration of stimulation increases, resulting in an increase in the EPSC [9]. Similarly, in EDLT, the channel conductivity, and EPSC increase as more mobile protons inside the casein electrolyte accumulate at the electrolyte/channel interface during longer spike widths [32].

**Figure 5:**
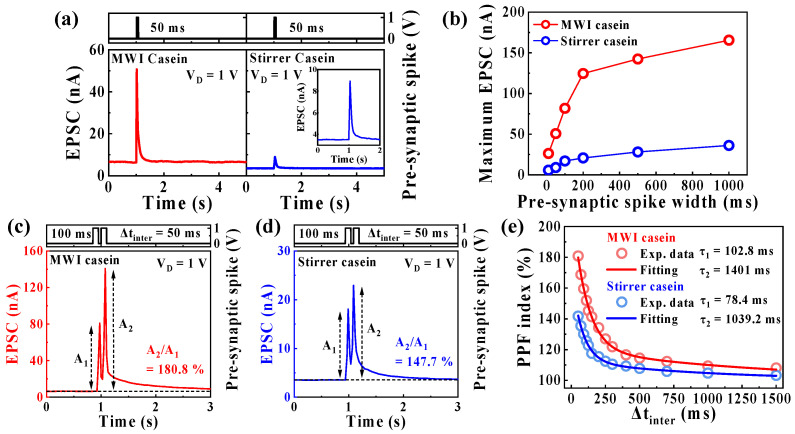
(**a**) Excitatory post-synaptic current (EPSC) of MWI and heat-stirrer synthetic casein electrolyte-gated EDLTs triggered by the pre-synaptic spike (1 V, 50 ms) at V_D_ = 1 V. (**b**) Maximum EPSCs with different pre-synaptic spike widths (10, 50, 100, 200, 500, and 1000 ms). EPSC facilitated by a paired pre-synaptic spike (1 V, 100 ms) with a 50 ms spike interval (Δt_inter_) of (**c**) MWI and (**d**) heat-stirrer synthetic casein electrolyte-gated EDLT. (**e**) Paired pulse facilitation index of MWI and heat-stirrer synthetic casein electrolyte-gated EDLTs as a function of Δt_inter_ from 50 to 1500 ms of the pre-synaptic spikes.

Figure 5a shows EPSCs of MWI and heat-stirrer synthetic casein electrolyte-gated EDLTs triggered by a single pre-synaptic spike with a spike amplitude of 1 V for a spike width of 50 ms at a V_D_ of 1 V. EPSCs for differences in pre-synaptic spike widths are shown in Figure S2 (Supplementary Materials). Figure 5b shows the maximum EPSC values with various pre-synaptic spike widths. The maximum EPSC value of MWI synthetic casein EDLT, which increased with the increase in the pre-synaptic spike width, was higher than that of the heat stirrer. PPF is the essential characteristic of short-term synaptic plasticity and represents the neuronal facilitation event as a function of spike interval (Δt_inter_), in which the post-synaptic potentials evoked by the second pre-synaptic spike increase further when the second closely follows the previous one [27,53,54]. The first pre-synaptic spike induced the gathering of mobile protons at the electrolyte/channel interface. Then, a second spike is applied, where when Δt_inter_ is short, in addition to incomplete relaxation protons, mobile protons are continuously accumulated at the interface, increasing the channel conductivity. Figure 5c,d show EPSCs facilitated by two consecutive pre-synaptic spikes (1 V, 100 ms) with a Δt_inter_ of 50 ms for the MWI and heat-stirrer synthetic case EDLTs. EPSCs triggered by various paired pre-synaptic spikes with Δt_inter_ of 50–1500 ms are shown in Figures S3 and S4 (Supplementary Materials). Figure 5e shows the PPF index as a function of Δt_inter_ calculated as the ratio of the first (A_1_) and second (A_2_) EPSCs, where the PPF index increases with shorter Δt_inter_ but decreases with longer Δt_inter_. In particular, MWI synthetic casein EDLT exhibits a higher facilitation, with a PPF index of 180%, than that of the heat stirrer (PPF index of 141%) at a Δt_inter_ of 50 ms based on a stronger EDL gating effect. The calculated PPF index was fitted with the following double exponential decay relationship [55]:

(1) $$PPF\;index=A+C_{1}\exp\left(-\Delta t_{inter}/\tau_{1}\right)+C_{2}\exp\left(-\Delta t_{inver}/\tau_{2}\right)$$

where *A* is a constant value, *C*_1_ and *C*_2_ are the magnitudes of the initial facilitation, and τ_1_ and τ_2_ are the characteristic relaxation times. The values of τ_1_ and τ_2_ in the MWI synthetic casein were 102.8 and 1401 ms, respectively, and 78.4 and 1039.2 ms in the heat-stirrer synthetic casein, respectively. The results are nearly identical to biological synapses and indicate that the proposed device allows for subdivisions of the synaptic temporal scale into rapid and slow stages lasting tens and hundreds of milliseconds [53].

Table 2 shows the performance comparisons between the proposed MWI synthetic casein electrolyte-gated EDLT and various biopolymer-based EDLTs. In our MWI synthetic casein EDLT, the time of EDL synthesis is considerably reduced and the performances are superior (I_on_/I_off_ and PPF index) or comparable (Capacitance and SS).

**Figure 6:**
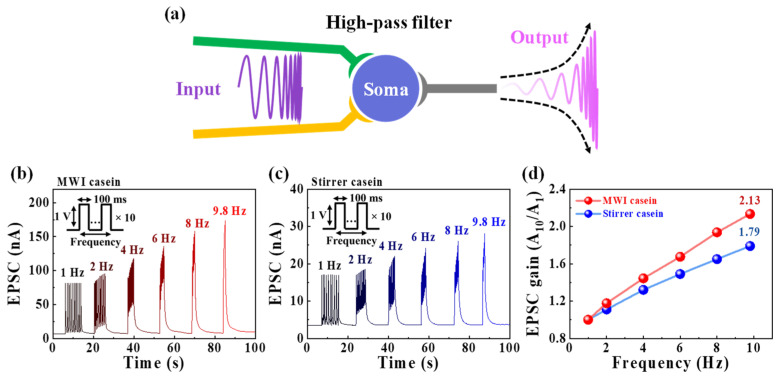
(**a**) Diagram of high-pass filtering function in biological synapses. EPSCs for 10 consecutive pre-synaptic spikes (1 V, 100 ms) at different frequencies (1 to 9.8 Hz) of (**b**) MWI and (**c**) heat-stirrer synthetic casein electrolyte-gated EDLT. (**d**) EPSC gain plotted against pre-synaptic spike frequency defined as A_10_/A_1_.

In addition, synapses perform the function of dynamically filtering information transactions based on short-term synaptic plasticity, as shown in Figure 6a [56]. Figure 6b,c show the EPSCs of MWI and heat-stirrer synthetic casein EDLTs in response to 10 successive pre-synaptic spikes with various frequencies from 1 to 9.8 Hz, respectively. Here, the rising and falling edge time of each pre-synaptic spike is 10 ns. EPSCs generated by sequential spikes remain almost constant at 1 Hz, increasing with progressively increasing frequency, indicating short-term facilitation. Thus, the high-pass temporal filtering functions of the EDLTs were successfully demonstrated [57]. Figure 6d shows the EPSC gain as a function of frequency, calculated from the ratio of dividing the EPSC-triggered 10th pre-synaptic spike (A_10_) by the EPSC-triggered first spike (A_1_). At a frequency of 1 Hz, the EPSC gain was 1 for both EDLTs; however, when the frequency was gradually increased to 9.8 Hz, the EPSC gain of the MWI synthetic casein EDLT rose to 2.13, which is greater than that of the heat stirrer (1.79). The MWI synthetic casein EDLT has a larger EPSC gain value based on higher facilitation than the heat stirrer, indicating that the high-pass filter can be operated over a wider gain range.

Synaptic weights are reinforced by repetitive stimuli to indicate long-term changes, which is long-term plasticity as opposed to short-term plasticity. Long-lasting strengthening or weakening of synaptic weights is considered long-term potentiation (LTP) or long-term depression (LTD) [58,59].

**Figure 7:**
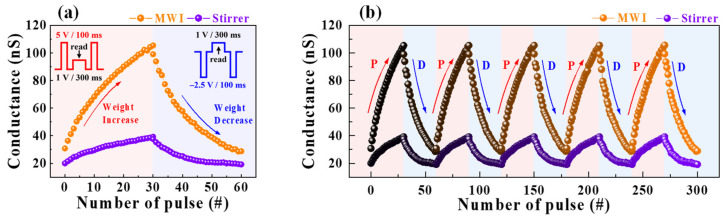
(**a**) Modulation of synaptic weights in potentiation and depression properties of MWI and heat-stirrer synthetic casein electrolyte-gated EDLTs by the number of the applied pre-synaptic spikes (#). Insets show schematic diagrams of reinforcement and depressive spikes. (**b**) Endurance test for five cycles of MWI and heat-stirrer synthetic casein electrolyte-gated EDLTs.

Figure 7a shows the modulation of synaptic weights in potentiation and depression by repetitive pre-synaptic stimuli, which consequently characterizes LTP and LTD. In both EDLTs, the channel conductance was changed by a potentiation pulse (5 V, 100 ms) and a depression pulse (−2.5 V, 100 ms). In MWI synthetic casein EDLT, the conductance increased from 30.7 to 105.4 nS for the potentiation stimulus, whereas it decreased from 99 to 28.7 nS for the depression stimulus. This indicates that the stimulation-dependent conductivity modulation of MWI is larger than that of the heat stirrer, which is 20 to 39.2 nS for the potentiation stimulus and 37.6 to 19.2 nS for the depression stimulus. The endurance characteristics of potentiation/depression in five cycles of potentiation and depression pulses are shown in Figure 7b, where the conductivity modulation was maintained as almost constant.

### 3.6. Recognition Simulation

**Figure 8:**
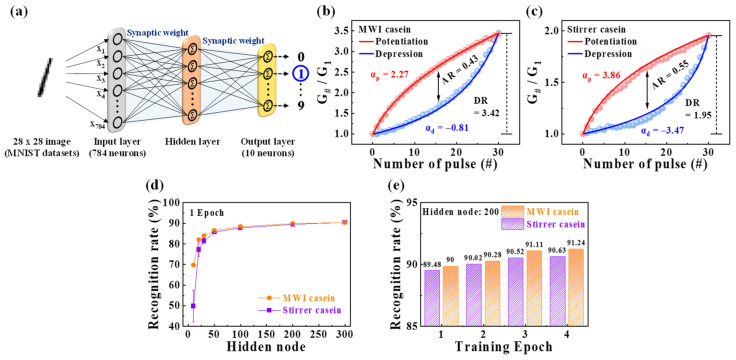
(**a**) Schematic diagram of an artificial neural network model with input, hidden, and output layers fully connected via synaptic weights for simulating MNIST recognition. Normalized potentiation and depression by nonlinearity analysis of (**b**) MWI and (**c**) heat-stirrer synthetic casein electrolyte-gated EDLTs. (**d**) Simulated MNIST recognition rates with varying numbers of hidden neurons. (**e**) Recognition rate by training epoch.

ANN is a computing architecture that performs complicated calculations, such as recognition and perception, with a complex network structure in which neurons are interconnected, similar to the human brain. To implement neuromorphic systems, it is essential to build ANNs based on synaptic devices in terms of hardware [60,61]. Figure 8a shows a designed multilayer ANN model in which the input, hidden, and output layers are fully connected via synaptic weights. The input and output layers were made up of 784 input neurons corresponding to 28 by 28 MNIST data and 10 output neurons related to the ten varieties of digits (0–9), respectively. This model was used for the handwritten MNIST learning test to evaluate the capability of neuromorphic computing. Figure 8b,c show the normalized potentiation and depression conductance of MWI and heat-stirrer synthetic casein EDLTs, respectively. The normalized conductance (G_#_/*G*_1_) was determined by dividing the conductance of each step (*G*_#_) by the first conductance (*G*_1_) and was used as a synaptic weight related to the synaptic strength connecting each neuron in the developed model. Through nonlinearity analysis of the normalized conductance, important characteristics, including dynamic range (DR), asymmetric ratio (AR), and linearity, which are highly correlated with the accuracy of learning and recognition, were obtained. The DR represents the range of conductance modulation defined by dividing *G*_max_ by *G*_min_. Higher DR values are required for better accuracy and performance in simulations [62]. The DR value of the MWI synthetic casein EDLT was 3.42, which was 1.75-times greater than that of the heat stirrer (1.95). The AR indicates the asymmetry of the changes in the potentiation and depression conductance. The more symmetrical the conductance modulation is, the closer the AR is to the ideal value of 0 and the higher the learning accuracy. The AR can be extracted using the equation [62]:

(2) $$\mathrm{AR}=\frac{max\left|G_{p}\left(n\right)-G_{d}\left(n\right)\right|}{G_{p\left(30\right)}-G_{d\left(30\right)}}\;\;\mathrm{for}\;\;n=1\;to\;30$$

The obtained ARs are 0.43 and 0.55 for MWI and heat-stirrer synthetic casein EDLT, respectively, indicating that the MWI is closer to the ideal value and the conductance change is symmetric. Moreover, the linearity of the increase and decrease in the conductance is critical for recognition simulation. The nonlinearity factors can be defined as [63]:

(3) $$G=\{\begin{array}{ccc} G_{min}\times {\left(\frac{G_{max}}{G_{min}}\right)}^{w} & , & if\;\alpha =0\\ {\left(\left(G_{max}^{\alpha }-G_{min}^{\alpha }\right)\times w+G_{min}^{\alpha }\right)}^{1/\alpha } & , & if\;\alpha \neq 0 \end{array}$$

where *G*_*max*_ and *G*_*min*_ are the maximum and minimum conductance, respectively, and w represents an internal variable ranging from 0 to 1. Further, α_p_ and α_d_ donate the nonlinearity factors for potentiation and depression, with an ideal value of 1. The calculated α_p_ and α_d_ of MWI synthetic casein EDLT were 2.27 and −0.81, which exhibited higher linearity in the increase and decrease in conductivity than that of the heat stirrer (α_p_ = 3.86, α_d_ = −3.47). Subsequently, an ANN model was designed using the normalized conductance and the obtained factors, trained with 60,000 MNIST images from the training dataset, and tested for recognition with 10,000 handwritten images from the test dataset in each training epoch. Figure 8d,e show the increase in the recognition rate with the increasing number of hidden nodes and training epochs, respectively. Based on the improved potentiation and depression characteristics and factors in the MWI synthetic casein, a higher recognition rate was achieved than that of heat-stirrer synthetic casein. The recognition rates of MWI and heat-stirrer synthetic casein EDLTs were 90% and 89.48%, with 200 hidden nodes, and 91.24% and 90.63% after four epochs, respectively. Therefore, the MWI synthetic casein EDLT represents potential as a promising artificial synapse compared to the heat stirrer.

## 4. Conclusions

We fabricated casein biopolymer electrolyte-gated EDLTs by introducing microwave-assisted synthesis to casein electrolyte solution preparation. Through MWI assistance, the synthesis time of the casein electrolyte was significantly reduced from 6 h to 5 min. Further, FT-IR analysis and C–*f* characterization on synthetic casein electrolytes demonstrated that the MWI processing had a higher EDL gating effect and ionic conductivity between the channel and gate than the conventional heat-stirrer processing. The MWI synthetic casein electrolyte-based EDLT exhibited superior double-swept transfer and output properties compared to the heat-stirrer synthetic casein electrolyte-based EDLT. Furthermore, the MWI synthetic casein EDLT successfully implemented improved plasticity functions of synaptic devices, such as EPSC, PPF, signal filter, and potentiation/depression, based on higher synaptic facilitation compared to the heat stirrer. Additionally, in the handwritten MNIST learning test, the MWI synthetic casein EDLT achieved higher recognition rates than the heat stirrer under the same learning conditions. Therefore, these results indicated that the casein electrolyte-gated EDLTs based on the microwave-assisted solution synthesis process with advanced characteristics are expected to play a key role in building bio-friendly neuromorphic computing systems, replacing conventional heat-stirrer synthesis.

## Figures and Tables

**Table 1:** Electrical parameters of MWI and heat-stirrer synthetic casein electrolyte-gated EDLTs.

EDL Type	I_on_/I_off_ (A/A)	V_th_ (V)	ΔV (V)	SS (mV/dec)	μ_FE_ (cm^2^/V·s)	D_it_ (cm^2^·eV^−1^)
MWI casein	4.31 × 10^7^	0.61	2.51	131.59	18.62	1.88 × 10^11^
Heat stirrer casein	4.12 × 10^6^	0.99	1.75	332.87	2.24	2.03 × 10^11^

**Table 2:** Performance comparison of this EDLT with other biopolymer EDL-based EDLTs.

Biopolymer EDL	Synthesis Condition	Capacitance at 1 Hz	I_on_/I_off_	SS	PPF Index	Ref.
Starch	Heat-stirrer (90 °C, 30 min)	~1.5 μF/cm^2^	~1.6 × 10^6^ A/A	~140 mV/dec	~162% (Δt_inter_ = 10 ms)	[30]
Pectin	Heat-stirrer (70 °C, 30 min)	~3.7 μF/cm^2^	~6.6 × 10^5^ A/A	~196 mV/dec	~150% (Δt_inter_ = 10 ms)	[33]
PVA	Heat-stirrer (100 °C, 4 h)	~1.63 μF/cm^2^	~7.8 × 10^6^ A/A	~129.7 mV/dec	~123% (Δt_inter_ = 50 ms)	[54]
Casein	MWI 250 W (≈150 °C, 5 min)	~3.58 μF/cm^2^	~4.31 × 10^7^ A/A	~131.59 mV/dec	~180% (Δt_inter_ = 50 ms)	This work

## Data Availability

Not applicable.

## Supplementary Materials

The following supporting information can be downloaded at: https://www.mdpi.com/article/10.3390/polym15020293/s1, Figure S1: Thickness and surface roughness of spin-coated casein electrolyte films prepared through the MWI and heat stirrer; Figure S2: EPSCs triggered by single pre-synaptic spikes with different spike widths (10, 50, 100, 200, 500, 1000 ms) of MWI and heat-stirrer synthetic casein electrolyte-gated EDLTs; Figure S3: EPSCs facilitated by paired pre-synaptic spikes with various spike intervals from 50 to 1500 ms of MWI synthetic casein electrolyte-gated EDLT; Figure S4: EPSCs facilitated by paired pre-synaptic spikes with various spike intervals from 50 to 1500 ms of heat-stirrer synthetic casein electrolyte-gated EDLT.
