# Supplementary material for: Enhanced Synaptic Properties in Biocompatible Casein Electrolyte via Microwave-Assisted Efficient Solution Synthesis
Source: Polymers (Basel). 2023 Jan 6;15(2):293. doi: 10.3390/polym15020293 (PMC9864603; doi:10.3390/polym15020293)
Supplement: Supplementary file 1 [file polymers-15-00293-s001.zip › polymers-2110186-supplementary.pdf]

Supplementary Materials

Enhanced Synaptic Properties in Biocompatible Casein Electrolyte by Microwave-Assisted Efficient Solution Synthesis

Hwi-Su Kim[1], Hamin Park[2] and Won-Ju Cho[1,]*

[1]Department of Electronic Materials Engineering, Kwangwoon University, Seoul 01897, Korea

[2]Department of Electronic Engineering, Kwangwoon University, Seoul 01897, Korea

* Correspondence to: chowj@kw.ac.kr

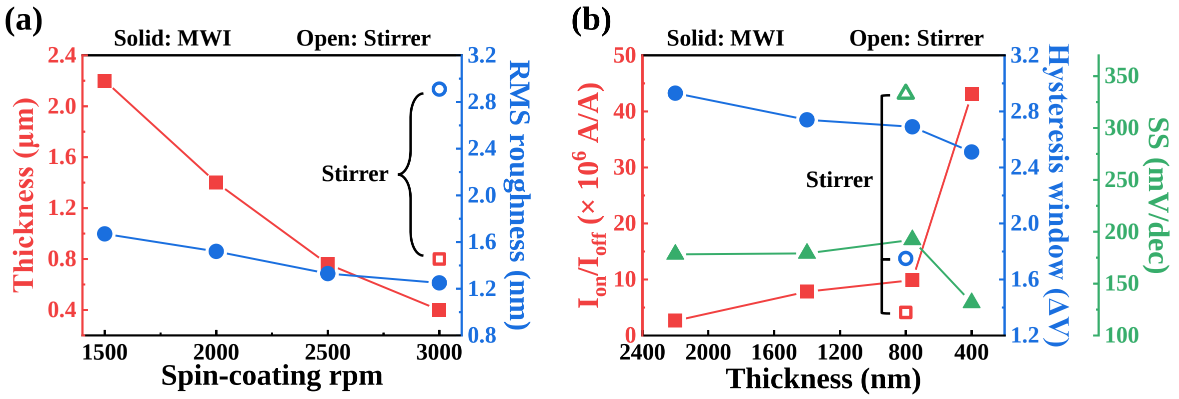

Figure S1. (a) Thickness and root-mean-square (RMS) roughness of MWI synthetic casein electrolyte film according to the spin-coating rpm for 30 s. These quantities were measured using a DektakXT Bruker stylus profiler. (b) Electrical parameters of MWI synthetic casein electrolyte-gated EDLT according to the thickness of the spin-coated casein EDL.

Figure S1(a) shows the thickness and surface roughness of the spin-coated MWI synthetic casein electrolyte film at different rpm for 30 s. At 3000 rpm, the thicknesses of MWI and heat-stirrer synthetic casein electrolyte film was approximately 400 nm and 800 nm, respectively. The thickness of the spin-coated MWI synthetic casein film at 2500 rpm was 760 nm, which is similar to that of the heat-stirrer film at 3000 rpm. The root-mean-square (RMS) roughness at 3000 rpm spin-coated MWI and heat-stirrer synthetic casein film was approximately 1.25 nm and 2.91 nm, respectively. The MWI synthetic casein films coated at 1500–3000 rpm exhibited a smoother surface condition with lower RMS roughness values than the heat-stirrer at 3000 rpm. And then, MWI synthetic casein electrolyte-gated EDLTs were fabricated, and their electrical characteristics according to the thickness were compared, as shown in Figure S1(b). As the thickness of the MWI synthetic casein electrolyte increased, the on/off current ratio (I_{on}/I_{off}) decreased, and the hysteresis window (ΔV) increased. As a result of comparing the electrical parameters of the MWI and heat-stirrer synthetic casein electrolyte-gated EDLTs with similar thickness, MWI synthetic casein EDLT had enhanced electrical characteristics with a higher I_{on}/I_{off}, larger ΔV, and smaller subthreshold swing (SS) than the heat-stirrer.

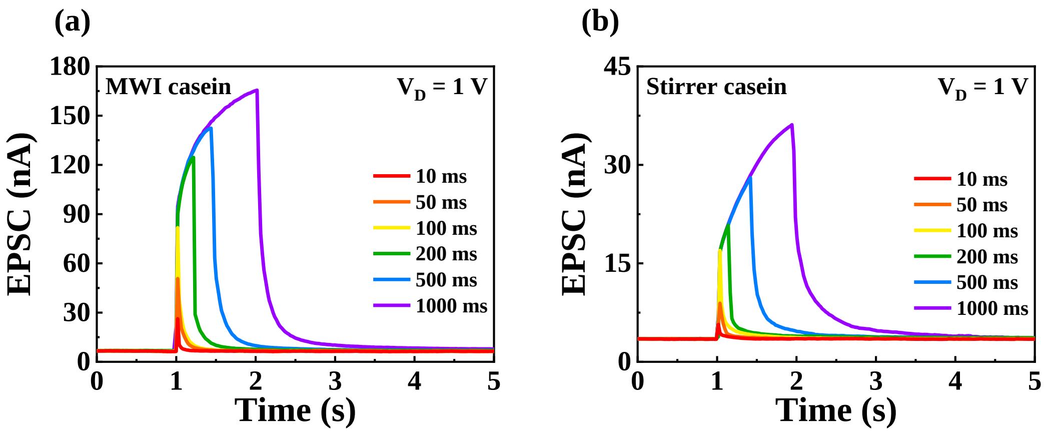

Figure S2. Excitatory post-synaptic current (EPSC) triggered by a single pre-synaptic spike of 1 V amplitude with different spike widths (10, 50, 100, 200, 500, and 1000 ms) at V_D = 1 V in (a) MWI and (b) heat-stirrer synthetic casein electrolyte-gated EDLTs.

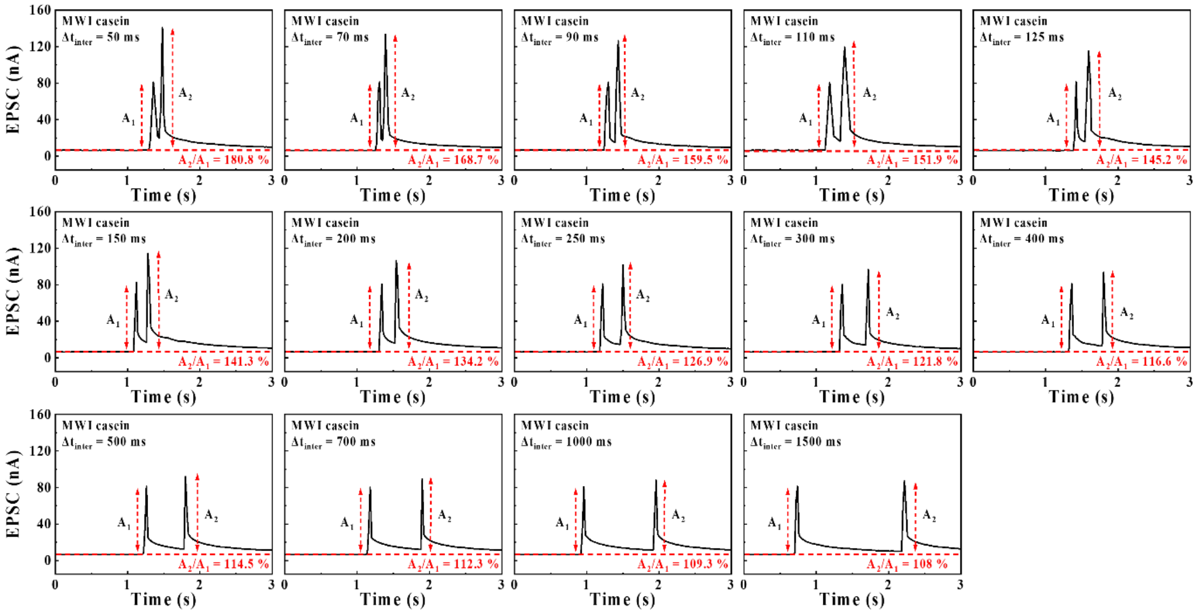

Figure S3. EPSCs facilitated by a paired pre-synaptic spike (1 V, 100 ms) with various spike intervals (Δt_{inter}) ranging from 50 to 1500 ms of MWI synthetic casein electrolyte-gated EDLT.

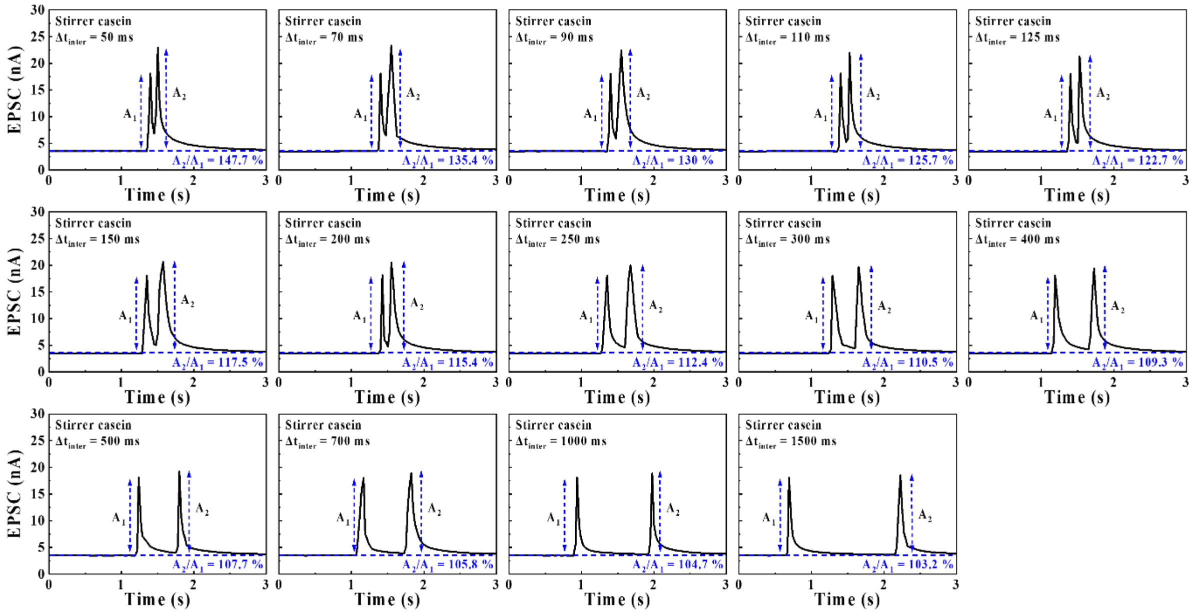

Figure S4. EPSCs facilitated by a paired pre-synaptic spike (1 V, 100 ms) with various spike intervals (Δt_{inter}) ranging from 50 to 1500 ms of heat-stirrer synthetic casein electrolyte-gated EDLT.
